# Acetylcholinesterase Inhibitors (AChEI's) for the treatment of visual hallucinations in schizophrenia: a case report

**DOI:** 10.1186/1471-244X-10-68

**Published:** 2010-09-07

**Authors:** Sachin S Patel, Azizah Attard, Pamela Jacobsen, Sukhi Shergill

**Affiliations:** 1 National Psychosis Unit, South London and Maudsley NHS Foundation Trust, Bethlem Royal Hospital, Monks Orchard Rd, Beckenham, BR33BX, UK; 2 Kings College London, Institute of Psychiatry, De Crespigny Park, London, SE5 8AF, UK

## Abstract

**Background:**

Visual hallucinations are commonly seen in various neurological and psychiatric disorders including schizophrenia. Current models of visual processing and studies in diseases including Parkinsons Disease and Lewy Body Dementia propose that Acetylcholine (Ach) plays a pivotal role in our ability to accurately interpret visual stimuli. Depletion of Ach is thought to be associated with visual hallucination generation. AchEI's have been used in the targeted treatment of visual hallucinations in dementia and Parkinson's Disease patients. In Schizophrenia, it is thought that a similar Ach depletion leads to visual hallucinations and may provide a target for drug treatment

**Case Presentation:**

We present a case of a patient with Schizophrenia presenting with treatment resistant and significantly distressing visual hallucinations. After optimising treatment for schizophrenia we used Rivastigmine, an AchEI, as an adjunct to treat her symptoms successfully.

**Conclusions:**

This case is the first to illustrate this novel use of an AchEI in the targeted treatment of visual hallucinations in a patient with Schizophrenia. Targeted therapy of this kind can be considered in challenging cases although more evidence is required in this field.

## Background

Visual hallucinations occur in a variety of neurological and psychiatric disorders and are prominent in the dementias and psychotic illness. The treatment of this distressing symptom often targets the underlying illness rather than the symptom. The pathophysiology of visual hallucinatory generation however remains unclear and more recent research has focused on acetylcholine depletion and its association with visual hallucinations.

To be able to better understand visual hallucinatory experience, we must first consider how normal cognitive processing enables the brain to process elementary visual stimuli and convert them into meaningful percepts. Bayesian statistical principles offer an elegant model on which to conceptualise the visual pathway. It is proposed that ascending stimulus driven and descending context driven pathways combine in an iterative manner to produce an accurate visual experience of our surroundings [1,2]. Acetylcholine is thought to play a pivotal role in modulating this pathway with low levels correlating to a greater degree of context driven visual representations and thus contextual inaccuracy [3]. This contextual inaccuracy could explain visual hallucinations as images would be perceived despite their absence in external space.

Diseases with significant Ach depletion include the dementias (in particular Lewy Body Dementia) and Parkinson's Disease. Drug therapies to increase levels of Ach are readily available (AchEI's) and there is evidence to suggest their efficacy in the treatment of visual hallucinations in these conditions [4-8] Utilising current models of visual hallucination generation and evidence for the use of AchEI's in related disorders it would appear that Ach depletion also plays a similar role in Schizophrenia.

We present below a case of a patient with treatment resistant schizophrenia presenting with distressing visual hallucinations who we successfully treated with an AchEI, Rivastigmine.

## Case Presentation

Mrs A is a 43 year old female with a diagnosis of schizoaffective disorder. She was transferred to the National Psychosis Unit, a tertiary referral in-patient service which specialises in the management of treatment resistant psychotic illness. On admission she presented as dishevelled, agitated, thought disordered and labile in mood. She expressed grandiose and paranoid delusions, 3^rd ^person auditory hallucinations and visual hallucinations of large wild cats. Negative features included apathy and withdrawal. Mrs A had little insight into her illness. These symptoms had persisted largely unchanged despite in-patient management and compliance with antipsychotic and mood stabilising medications for the previous 6 months. These visual experiences were evident during the day in clear daylight and consciousness, but worse at night when she was alone in her bedroom; on admission, she would choose to sleep in the corridor so as to avoid these creatures- and had been doing so for over 6 months.

Mrs A first became unwell with features of a schizoaffective disorder at the age of 19. Following treatment and discharge there was a period of relative stability over the next 20 years during which she was under the care of her local community mental health team (CMHT). At the age of 40, Mrs A was again admitted following a breakdown in her ability to function in the community due to deterioration in her mental state. Various treatment strategies were utilised during this period, including clozapine, following failure of combinations of other atypical antipsychotics and mood stabilisers. She had responded well to a combination of clozapine, aripiprazole and escitalopram in terms of a reduction in persecutory delusions and auditory hallucinations, however her visual hallucinations remained vivid. These had then taken greater prominence in Mrs A's mental state and this subsequently led to more subjective distress. Socially she was quite isolative and did not maintain any relations with family or friends Her presentation was not thought to be related to non compliance, drug and alcohol misuse or psychosocial stressors. Physical Investigations were unremarkable. MRI and EEG were reported as normal and blood indices including thyroid function tests, copper, caeruloplasmin and autoantibody screens were negative.

Mrs A's PANSS score on admission was 79 (p30, n15, g34) and MMSE was 30/30. The pharmacological management plan was to commence and maintain semi-sodium valproate within therapeutic plasma levels, reduce and discontinue her clonazepam and to restabilise on clozapine therapy. Following 4 months of this therapy with clozapine at a dose of 450 mg per day and in combination with psychological and occupational therapy, Mrs A's mental state stabilised with marked improvement in her delusions and auditory hallucinations, stable mood and better function. Her PANSS rating improved to a total score of 52 (p14, n13, g 25). Despite these improvements on clozapine, Mrs A continued to experience vivid visual hallucinations of tigers and lions.

A decision was made by the multidisciplinary team to begin an AChEI, Rivastigmine to target visual hallucination symptoms. Rivastigmine patches at 4.6 mg/24 hrs was initiated. No changes were made to all other psychotropic medications. PANSS rating scales and MMSE scores were done on two occasions following the addition of rivastigmine patches to therapy. In addition a tailored visual hallucination rating scale was developed, adapted from the Psychotic Symptom Rating Scales for auditory hallucinations (PSYRATS) [9]. This consisted of 3 items measuring frequency, vividness and distress associated with the hallucinations. Items were scored 0-4 (frequency and distress) or 0-3 (vividness), and were clinician-rated. Ratings were taken daily by the primary or allocated nurse in the two weeks prior to treatment with rivastigmine and during treatment.

Mrs A continued to show an improvement in her functioning, demonstrated by the fact she now slept consistently in her own bedroom at night, was independent in her self-care, and started to participate in community outings and OT activities. Mrs A's level of occupational and psychological therapy input remained stable throughout the introduction of rivastigmine patches, and focused on reducing the distress and interference with daily activities associated with the visual hallucinations. No further medication changes were made to her pharmacological therapy. Mrs A suffered no untoward side effects from the rivastigmine patches. Two weeks following the addition of rivastigmine patches her PANSS total score was 45 (P 13, N 10, GP 22) and this improvement was maintained as her PANSS total score at 7 weeks was 43 (P 11, N 10, GP 22). Over the baseline assessment period, Mrs A continued to report distressing visual hallucinations throughout the day. After the rivastigmine treatment was initiated, after 3 weeks of treatment, reporting of visual hallucinations was decreased to once a day on average, and the level of distress was significantly reduced. This one appearance a day was usually reported as seeing a lion or tiger in her bedroom when she woke up in the middle of the night. Slowly, even this report became much more ambiguous; the animals were more unclear at night and she had more difficulty making them out. Subjectively, Mrs. A reported that she thought the patches were helpful and she was seeing the animals less frequently than before. She was discharged from hospital and at 6 month follow up was living independently quite successfully, with support from her locality mental health team, and remaining free from visual hallucinations and continuing her rivastigmine.

## Conclusions

This case illustrates a novel use for AchEI's in the targeted treatment of visual hallucinations in Schizophrenia. Often the most challenging cases faced in clinical psychiatry are those with treatment resistant symptoms which can prove distressing to patients. Our approach in this case was to combine current thinking in neurophysiology and therapeutic evidence in related disorders and then to apply these to clinical practice in a targeted way. We appreciate that this is a single case and a novel therapeutic use however we feel that further research in this field is indicated.

## Competing interests

Authors have no competing interests to declare that are relevant to the content of this submission.

## Authors' contributions

SS contributed to planning, supervision and writing the report. SP reviewed the literature. SP, PJ and AA each contributed to writing the case presentation.

## Consent

Written informed consent was obtained from the patient for publication of this case report. A copy of the written consent is available for review by the Editor-in-Chief of this journal.

## Pre-publication history

The pre-publication history for this paper can be accessed here:

http://www.biomedcentral.com/1471-244X/10/68/prepub
